# Change in brain volume and cortical thickness after behavioral and surgical weight loss intervention

**DOI:** 10.1016/j.nicl.2018.101640

**Published:** 2018-12-10

**Authors:** Cara Bohon, Allan Geliebter

**Affiliations:** a Department of Psychiatry and Behavioral Sciences, Stanford University School of Medicine, Stanford, CA, USA; b Department of Psychiatry, Icahn School of Medicine at Mount Sinai, Touro College and University System, New York, NY, USA; c Department of Psychology, Touro College and University System, New York, NY, USA

**Keywords:** Weight loss, Bariatric surgery, Cortical thickness, Gray matter volume, White matter volume

## Abstract

Obesity is associated with reduced cortical thickness and brain volume, which may be related to poor nutrition. Given that brain atrophy in anorexia nervosa recovers with nutritional improvements and weight gain, it is worth examining how brain structure changes at the other end of the weight spectrum with weight loss. Thus, this study aimed to examine change in cortical thickness and brain volume in 47 patients with severe obesity who participated in no treatment, behavioral weight loss, or bariatric surgery. T1-weighted MRI scans were conducted pre-treatment and approximately four months later. Measures of cortical thickness, gray matter volume, and white matter volume were compared between time points. Despite overall reduction in BMI, there was no significant change in cortical thickness. There was a significant increase in left hemisphere gray matter and white matter volumes across the sample. At baseline and follow-up, there was no relationship between cortical thickness or brain volumes and BMI. This study is the first to examine changes in cortical thickness and brain volume with weight loss in adults with obesity and the findings show partial support for the hypotheses that weight loss results in increased cortical gray and white matter.

## Highlights

• Left hemisphere gray and white matter volume increased over 4-months in patients with severe obesity who were losing weight.
• Change in BMI was not related to change in brain volume or cortical thickness.
• There were no relationships between BMI and cortical thickness or gray or white matter volumes at baseline or follow-up.

## 1 Introduction

Obesity has become a major health concern affecting about one-third of the population (Flegal et al., 2016). Studies reveal negative correlations between body mass index (BMI) and brain volume (Debette et al., 2010; Walther et al., 2010; Ward et al., 2005) and cortical thickness (Veit et al., 2014). Additionally, studies show reduced gray matter volume (Gunstad et al., 2008; Pannacciulli et al., 2006; Raji et al., 2010; Yokum et al., 2012) and reduced cortical thickness in individuals with obesity compared to those of normal weight (Marqués-Iturria et al., 2013). There are no established mechanisms for this association. However, on the other end of the weight spectrum, underweight patients with anorexia nervosa, a psychiatric illness characterized by severe food restriction and malnutrition, also show reduced brain volume in both gray matter and white matter (Katzman et al., 1996; Seitz et al., 2016; Titova et al., 2013), which improves with recovery and weight regain (Lambe et al., 1997; Seitz et al., 2016; Swayze et al., 2003; Wagner et al., 2006). Cortical thinning is also present in acute anorexia nervosa, and normalizes after weight restoration (Bernardoni et al., 2016; King et al., 2015). Thus, the correlations between brain volume and obesity may also be related to nutritional status.

Eating in excess of daily energy requirements is a primary factor in weight gain (Tataranni, 2000). This excess may result from overall increased intake of food or increased intake of calories via greater proportion of high energy-dense foods, thus changing the balance of nutrient intake (Xanthakos, 2009). Overnutrition and thus overweight status can have direct and indirect effects on brain structure and function (Conklin et al., 2007; Eyles et al., 2003).

Thus, given reduced brain volumes and cortical thickness in individuals who are both over- and underweight, it is important to assess how weight loss via treatment for obesity may alter brain structure.,A small study examining cortical thickness in five patients before and after bariatric surgery revealed a large, but non-significant positive relationship (R^2^ = 0.578 for right and R^2^ = 0.617 for left hemisphere) between increase in global cortical thickness and percent excess weight loss (Bohon et al., 2018) suggesting that change toward a healthy weight was associated with improvements in cortical thickness. Because that study only followed five patients, the present aims were to examine change in cortical thickness and brain volume with weight loss in an existing larger sample of adult patients with severe obesity, before and after one of three conditions: no treatment, behavioral weight loss program, or bariatric surgery. Although these data did not include measures of nutritional status we were interested in the differences between groups given the assumed differences in food intake for each group.

## 2 Method

### 2.1 Participants and procedure

The sample included 50 adults with extreme obesity (mean BMI = 42.35, range 37–53). All surgical participants were recruited from the Center for Weight Loss Surgery at a large university-affiliated hospital in New York City after they qualified for bariatric surgery. Comparably obese subjects were recruited to enroll in a behavioral weight loss program in conjunction with a low-calorie liquid formula diet or have no treatment. This resulted in 20 adults receiving either Roux-en-Y gastric bypass (*n* = 12) or sleeve gastrectomy (*n* = 8), 14 receiving behavioral weight loss, and 16 receiving no treatment, totaling 50. Of these, there were usable neuroimaging data at 4-month follow-up for 47 participants. (The three participants without follow-up data all received sleeve gastrectomy). The sample was 86% female, 40% Hispanic/Latinx, and by race, 44% Black/African American, 12% White/Caucasian, 4% Asian/Pacific Islander, and 40% other/more than one race. Participants were right-handed, nonsmoking, and free of any major psychiatric or physical disorders (including diabetes) and were not taking any medication that may have affected body weight. Institutional review boards at Columbia University and St. Luke's Roosevelt Hospital approved the study, and all participants provided informed consent.

Participants were assessed prior to treatment and approximately 4 months afterward. Structural MRI scans occurred between 11 am and 1 pm after an overnight (12h) fast and 1.5 h after a standardized liquid meal (250 kcal). Time of day of the scan was consistent from baseline to follow-up for each participant. Following the structural MRI scans, participants also underwent functional MRI scans, during which visual and auditory cues of foods and nonfoods were presented, but those results are not reported here.

### 2.2 MRI acquisition

Scans were conducted on a 1.5 T twin-speed GE scanner with quadrature RF head coil. The participants were all able to fit into the scanner, which had a bore diameter of 60 cm. Motion was minimized by placing pads around the head and tape across the forehead. Participants were in the scanner for approximately 60 min. T1-weighted high-resolution (1 mm voxel) scans were performed. The acquisition parameters were: echo time = 60 ms, repetition time = 4 s, flip angle = 60°.

### 2.3 Data analysis

Structural T1-weighted images were processed with FreeSurfer 5.3.0 software (http://surfer.nmr.mgh.harvard.edu). The standard FreeSurfer cortical reconstruction process was used, which includes motion correction, transformation, intensity correction, skull stripping, volume labeling, segmentation, surface extraction and modeling, cortical thickness estimation, registration, and parcellation. Technical details of these procedures are described in prior publications (Dale et al., 1999; Fischl and Dale, 2000; Fischl et al., 1999; Fischl, Salat et al., 2004; Fischl, van der Kouwe, et al., 2004; Fischl et al., 2002). Calculation of cortical thickness, the primary measure of interest, followed procedures validated against histological analysis (Rosas et al., 2002) and manual measurements (Kuperberg et al., 2003; Salat et al., 2004). Global measures of cortical thickness, gray matter volume, white matter volume, and intracranial volume were extracted from the results of the FreeSurfer procedures after being quality checked by the first author.

Brain measures were imported into SPSS Version 23, which was used to conduct repeated measures ANOVAs to examine the effect of time on change in cortical thickness, gray matter volume, and white matter volume, with treatment condition as a fixed between subject factor to explore the differences that treatment had on brain structure given different nutritional intakes for each group. Regression analyses were also conducted to examine the relationship between BMI and brain measures at baseline as well as at follow-up in an attempt to replicate prior correlations between obesity and brain volume and cortical thickness. Finally, we conducted regression analyses examining the relationship between change in BMI and change in brain volumes. These planned analyses result in 12 statistical tests (3 repeated measures ANOVAs and 9 regressions). Thus, alpha was set to *p* = .004 (0.05/12) using Bonferroni standards to correct for multiple comparisons. We utilized global measures of volume and thickness because the most robust and consistent effects in prior research were in relation to global measures, rather than regional.

## 3 Results

There was a significant reduction in BMI across the sample (F(1,44) = 687.6, *p* < .001) as well as a group by time interaction (F(2,44) = 167.8, *P* < .001). Marginal means are displayed in Table 1. Each group, including the no treatment group, showed a significant reduction in BMI, with the surgery group showing the largest reduction, followed by the behavioral weight loss group, and the no treatment group, with the smallest reduction. Post hoc independent samples *t*-tests between each group on the change in BMI revealed significant differences between the no treatment group and the behavioral weight loss group, between the no treatment group and the surgery group, and between the behavioral weight loss group and the surgery group.

**Table 1:** Baseline (T1) and follow-up (T2) assessments within the three groups (Means ± SE).

	No treatment (*n* = 16)	Behavioral weight loss (*n* = 14)	Bariatric surgery (*n* = 17)
T1 BMI	41.19 ± 0.94	42.71 ± 1.00	43.77 ± 0.91
T2 BMI	40.43 ± 1.00	37.50 ± 1.07	34.65 ± 0.97
T1 LHCT	2.25 ± 0.28	2.21 ± 0.30	2.27 ± 0.28
T2 LHCT	2.25 ± 0.28	2.20 ± 0.30	2.26 ± 0.27
T1 RHCT	2.24 ± 0.36	2.20 ± 0.38	2.25 ± 0.35
T2 RHCT	2.26 ± 0.30	2.20 ± 0.32	2.28 ± 0.29
T1 LHGM	199,180 ± 4873	199,428 ± 5210	201,122 ± 4728
T2 LHGM	203,135 ± 4979	202,074 ± 5322	203,563 ± 4830
T1 RHGM	201,993 ± 5187	201,403 ± 5545	201,537 ± 5032
T2 RHGM	200,322 ± 5029	200,100 ± 5376	203,149 ± 4879
T1 LHWM	219,683 ± 6863	221,881 ± 7337	216,556 ± 6658
T2 LHWM	224,098 ± 7082	223,694 ± 7571	218,625 ± 6870
T1 RHWM	220,777 ± 7284	223,656 ± 7787	217,768 ± 7067
T2 RHWM	223,004 ± 7173	221,919 ± 7668	217,414 ± 6958

Abbreviations: BMI: body mass index (in kg/m^2^); LHCT: left hemisphere cortical thickness (in mm); RHCT: right hemisphere cortical thickness (in mm); LHGM: left hemisphere gray matter volume (in mm^3^); RHGM: right hemisphere gray matter volume (in mm^3^); LHWM: left hemisphere white matter volume (in mm^3^); RHWM: right hemisphere white matter volume (in mm^3^).

There were three outliers in these assessment data, lying outside of the inner Tukey fences (calculated as the value of the first or third quartile ±1.5 times the interquartile range, using boxplots in SPSS), with two outliers for change in white matter volume and one outlier for change in gray matter volume. We excluded these outliers from relevant analyses. Repeated measures analyses revealed no significant effects of time or group on change in cortical thickness. There were also no group by time interaction effects on cortical thickness. For gray matter volume, however, there was a main effect of time on left hemisphere gray matter volume (F(1,43) = 24.92, *p* < .001, partial eta-squared = 0.37) with an increase in left hemisphere gray matter over time. For white matter volume, there was also a main effect of time on left hemisphere white matter volume (F(1,44) = 18.365, p < .001, partial eta-squared = 0.29), such that white matter also increased over time. There were no group by time interaction effects on gray or white matter volumes.

**Fig. 1:**
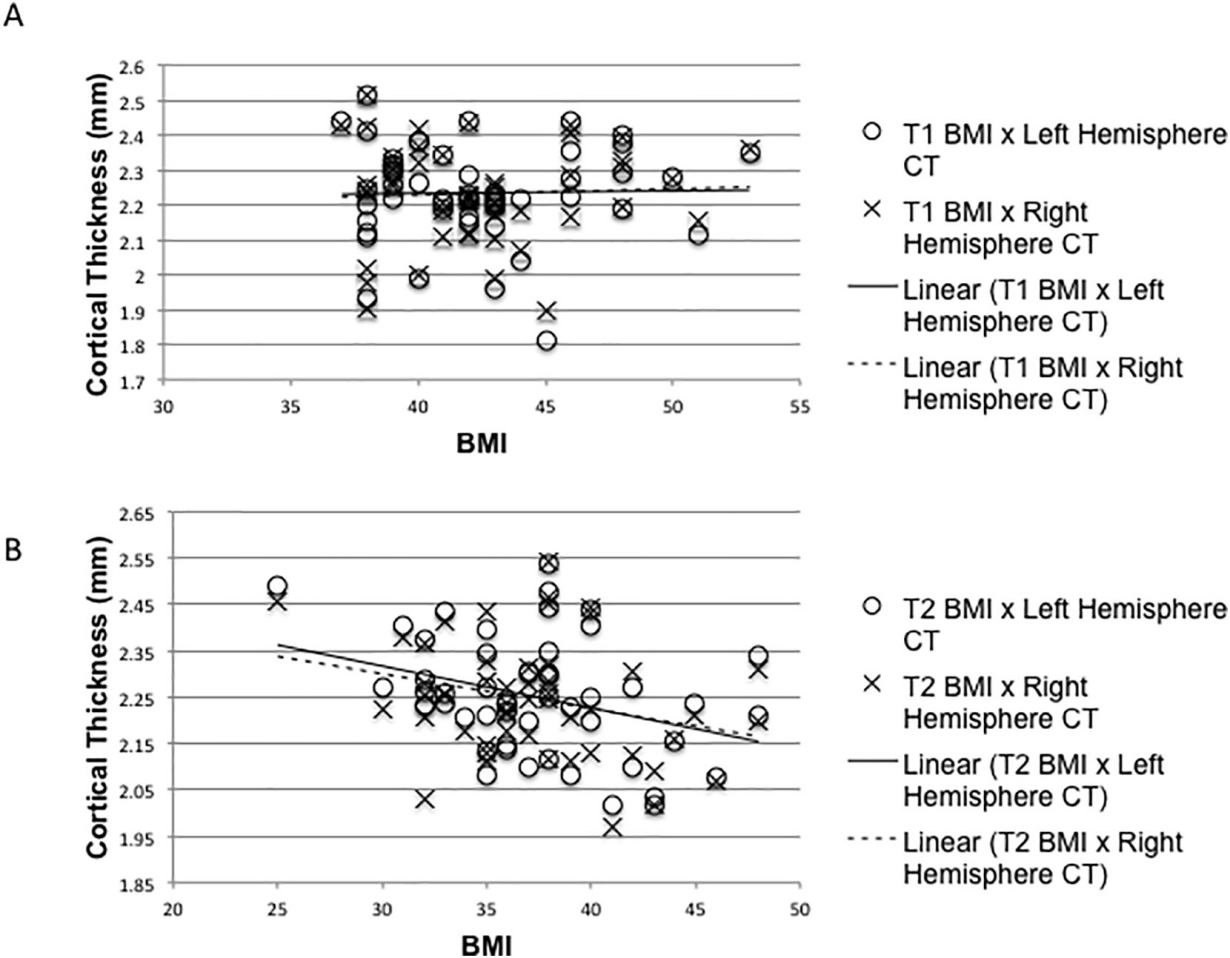
Relationship between cortical thickness (CT) and BMI at T1 (Panel A) and T2 (Panel B). The results show a nonsignificant negative correlation (left hemisphere: *r* = −0.33, p = .017, right hemisphere: *r* = −0.28, p = .051) between cortical thickness and BMI at T2 when there was a wider range in BMI that stretches into the normal weight range, but no correlation at T1 (left hemisphere: *r* = 0.02, *p* = .89, right hemisphere: *r* = 0.05, *p* = .73). Note that the ranges on each of the axes differ on the two graphs.

Regression analyses showed a nonsignificant inverse relationship between cortical thickness and BMI at follow-up (left hemisphere: Beta = −0.333, *p* = .017, R^2^ = 0.111; right hemisphere: Beta = −0.275, *p* = .051, R^2^ = 0.076), but not at baseline (Fig. 1). No significant correlations were found between white or gray matter volume and BMI at baseline or follow-up.

Finally, regression analyses were conducted to examine the relationship between changes in brain volume and cortical thickness and percentage change in BMI. These analyses did not reveal any significant effects.

Post-hoc paired samples *t*-tests also revealed no differences in change in volume or cortical thickness between hemispheres across the sample. Additionally, a post-hoc ANOVA revealed no effect of group or time on white matter hypointensities, which are thought to reflect tissue pathology.

## 4 Discussion

This study is the first to examine change in brain structure longitudinally in a sample of adults with severe obesity undergoing various weight loss interventions. Despite significant reductions in BMI, most notably in the group receiving bariatric surgery, there was no significant change in cortical thickness over time in the sample. However, there was a significant increase in left hemisphere gray matter and white matter volumes. Because most participants lost some amount of weight during the study, this change in volume could relate to weight loss. However, post hoc linear regressions examining the relationship between change in gray matter and white matter volumes and change in BMI did not find a significant effect, which does not suggest that change in BMI mediates the effect of time.

Thus, our findings show some support for our hypotheses that brain volume increases over time with weight loss in patients with severe obesity, although not for cortical thickness and restricted to the left hemisphere. Degree of change in BMI was also not significantly related to degree of change in volume, suggesting that weight change in general may result in increases in volume. Further, there were no group by time interactions, suggesting that nutritional status was not directly related to brain structural changes, but we did not have direct data on nutritional intake in these patients.

Prior research has been limited by the cross-sectional nature of studies, making this longitudinal study showing changes within participants over time an advance in our understanding of the relationship between body weight and brain structure. The lack of differences between treatment groups may be due to inadequate sample sizes per group and the variation in mean BMI reduction among the groups. Most participants lost at least some weight (including the no treatment group possibly because they were enrolled in an obesity study), but participants in the surgery group lost much more weight than other groups. Thus, these findings reflect brain change in a sample with minimal to marked weight loss. Given that dietary intake was likely very different between groups, the lack of group differences could suggest that dietary intake does not have as large an impact on brain volume change as hypothesized.

Prior research has shown that gray matter volume is more closely related to brain surface area than to cortical thickness (Winkler et al., 2010), which may help explain why cortical volume and thickness showed discrepant results in these analyses. Additionally, there is some evidence of inflammatory markers correlating with brain volumes (Jefferson et al., 2007), as well as evidence of heightened inflammation in obesity (Esposito et al., 2003). These markers may be worth exploring in the future as a possible mechanism for change in volume with weight loss.

Our findings were present only in the left hemisphere, but it is worth noting that there were no statistically significant differences in volume change between hemispheres. Although there are no known mechanisms that explain change in brain volume in one hemisphere only, there is evidence that the left hemisphere is impacted earlier and more severely in Alzheimer's disease (Toga and Thompson, 2003). Future research may help to better understand why lateralization in structural change occurs in some individuals, as it may have implications for cognitive functions, which are known to be impacted in obesity (Smith et al., 2011).

Prior evidence of a relationship between cortical thickness and brain volume with BMI was not replicated at baseline, and the correlation was not significant at follow-up. This may be due to low power or also may be due to the high BMIs or restricted range of the sample. Prior research showing significant correlations including a full range of healthy BMIs along with obese, so it may be that when BMI drops below a certain ceiling level or the range increases, that the linear relationship returns.

Although this study is the first to examine change in brain volume and cortical thickness after weight loss surgery or behavioral weight loss treatment, it is not without limitations. The cell sizes for the groups were small, limiting power to detect differences between the groups. Since dietary intake is a possible mechanism of change in brain structure during weight loss, it is reasonable to expect group differences given different dietary guidance in the no treatment, weight loss treatment, and surgery groups. However, the study lacks detailed data on dietary intake over the follow-up period. Information about intake of various nutrients involved in brain health could also help us better understand brain changes.

In sum, these results provide preliminary evidence that left hemisphere brain volume, although not cortical thickness, increases over time in a sample of adults with severe obesity. Most of the sample was in some form of weight loss treatment (bariatric surgery or behavioral weight loss treatment), and most participants lost weight during the study, although there were no effects of treatment group on the results. The results are complementary to evidence in samples of patients with anorexia nervosa, who show increases in volume and cortical thickness with weight gain, as it suggests that different ends of the weight spectrum respond similarly with changes in weight toward a normal range. Future research may be able to provide evidence of mechanisms for these effects by examining nutritional intake and utilizing larger samples.

## Sponsor

National Institutes of Health grant R01 DK080153 (Dr. Geliebter). Dr. Bohon's effort was supported by National Institute of Mental Health grant K23 MH10679401.
